# An In Vitro Evaluation of the Biological Effects of Carbon Nanotube-Coated Dental Zirconia

**DOI:** 10.1155/2013/296727

**Published:** 2013-08-20

**Authors:** Wen Kou, Tsukasa Akasaka, Fumio Watari, Göran Sjögren

**Affiliations:** ^1^Dental Materials Science, Department of Odontology, Faculty of Medicine, Umeå University, 90781 Umeå, Sweden; ^2^Department of Biomedical Materials and Engineering, Graduate School of Dental Medicine, Hokkaido University, Sapporo 060-8586, Japan

## Abstract

The purpose of this study is to evaluate functionalized multiwalled carbon nanotubes (fMWCNTs) as a potential coating material for dental zirconia from a biological perspective: its effect on cell proliferation, viability, morphology, and the attachment of an osteoblast-like cell. Osteoblast-like (Saos-2) cells were seeded on uncoated and fMWCNT-coated zirconia discs and in culture dishes that served as controls. The seeding density was 10^4^ cells/cm^2^, and the cells were cultured for 6 days. Cell viability, proliferation and attachment of the Saos-2 cells were studied. The results showed that Saos-2 cells were well attached to both the uncoated and the fMWCNT-coated zirconia discs. Cell viability and proliferation on the fMWCNT-coated zirconia discs were almost the same as for the control discs. Better cell attachment was seen on the fMWCNT-coated than on the uncoated zirconia discs. In conclusion, fMWCNTs seem to be a promising coating material for zirconia-based ceramic surfaces to increase the roughness and thereby enhance the osseointegration of zirconia implants.

## 1. Introduction

During the last few years, the popularity of dental zirconia implants has increased because they are tooth colored, biocompatible and have an osseointegration ability comparable to dental titanium implants [1, 2]. A fractography study by Gahlert et al. (2012) [1], however, indicates that the fracture initiation site of dental zirconia implants is often located to the stress concentration area in the thread; the grooves on the implant surface created by sandblasting often lead to stress concentration due to their notch effect. The purpose of sandblasting is to increase the surface area and roughness of the dental zirconia implant and thus improve osseointegration [3]. Sandblasting can, however, introduce defects on the surfaces of zirconia implant, which will act as potential fracture initiation sites [1]. The survival of dental zirconia implants should, therefore, improve if methods other than sandblasting could be used.

In 1991 carbon nanotubes (CNTs) were discovered by Iijima [4]. This material has been shown to have a large surface area, good mechanical strength, ultra-light weight, and excellent chemical and thermal stability [5]. The nanotubes are structures of single or multiple sheets of graphene rolled up to form single-walled carbon nanotubes (SWCNTs) and multiwalled carbon nanotubes (MWCNTs). Since their discovery CNTs have been used in many fields, such as in electrical and mechanical applications and for biological and medical purposes [6]. The lack of solubility in aqueous media has been a major technical barrier in biological and biomedical applications, but the recent development of methods to chemically modify and functionalize CNTs has made it possible to dissolve and disperse CNTs in water, making them more suitable materials for biological applications [7]. 

The disadvantage of sandblasting mentioned previously makes it of interest to investigate other techniques to increase the surface roughness of zirconia implants. One conceivable method could be the application of CNTs as a surface coating on the implants. In a survey of the literature, no paper was found addressing evaluation of the biological effects of the application of MWCNT coating to dental zirconia. The aim of the present study, therefore, was to evaluate in vitro functionalized MWCNTs as a coating material for dental zirconia from a biological perspective: its effect on cell proliferation, viability, morphology, and the attachment of an osteoblast-like cell.

## 2. Materials and Methods

### 2.1. Preparation of MWCNTs

MWCNTs used in this study were obtained from NanoLab (Brighton, MA, USA). According to the manufacturer, the MWCNTs were about 15 ± 5 nm in diameter and 1–5 *μ*m in length and produced by chemical vapor deposition method. The purity of the MWCNTs was about 95 wt%, and they were purified according to the method devised by Sato et al. [8]. The MWCNTs were later functionalized by carboxylation (MWCNT-COOH) to improve their dispersion in aqueous solution, according to Peng et al. [9]. 

### 2.2. Specimen Preparation

**Figure 1:**
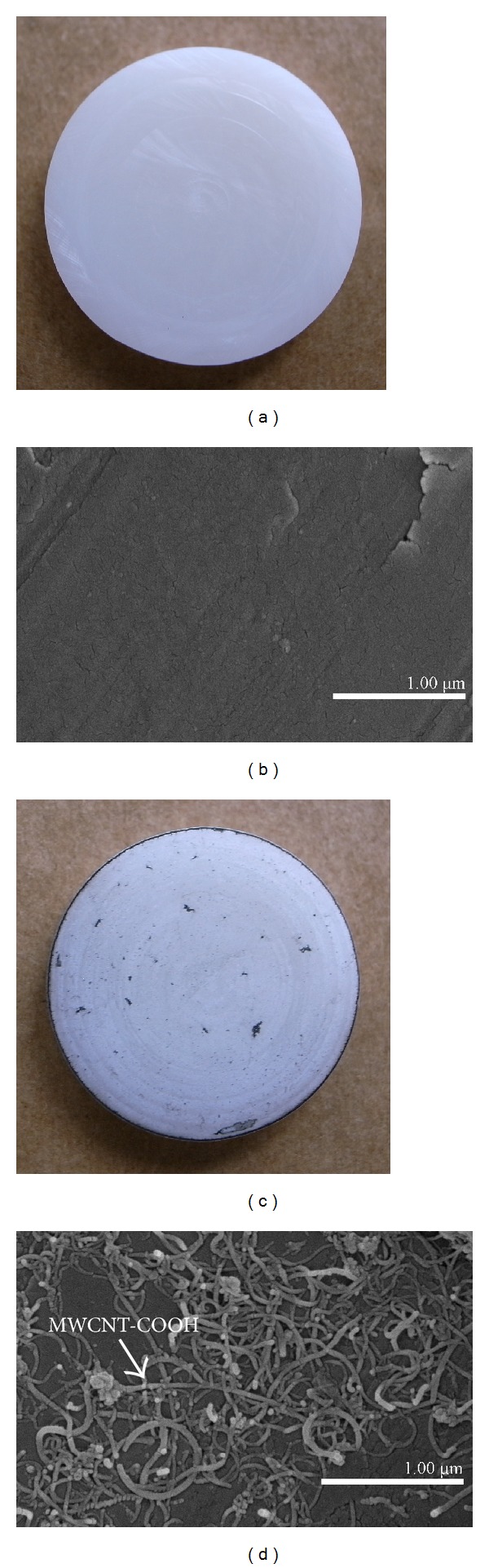
Uncoated (a, b) and MWCNT-COOH-coated (c, d) zirconia disc samples. (a, c) Macroview. (b, d) SEM images. A good covering of MWCNT-COOH can be observed on the coated zirconia discs (d).

Sixteen hot isostatic pressed (HIPed) yttria stabilized zirconia polycrystal (Y-TZP) discs (*⌀*13 mm, thickness: 2 mm) were prepared (Figure 1) by computer-aided design/computer-aided manufacturing (CAD/CAM) technique (CAD.esthetics AB, Skellefteå, Sweden). The HIPed Y-TZP discs were cleaned ultrasonically (Elma, Transonic 460/H, Singen, Germany) in deionized water for 30 min. The MWCNT-COOH were dispersed in 99.5% ethanol to a final concentration of 100 ppm with sonification (Sonicator Vibracell VC 130, 130 W, 20 kHz and amplitude 60% for 10 min). The MWCNT-COOH suspension obtained (1 mL/dish) was poured onto the zirconia discs and kept at room temperature inside a fume hood for 1 h. After the ethanol had evaporated, the discs were rinsed with deionized water and dried in a heated oven at 65°C for 20 min. Thereafter, the specimens were individually packaged in sterilization bags and sterilized in a steam autoclave (Tomy, BS-235, Tokyo, Japan) at 121°C for 15 min and dried in a heated oven at 65°C for 1 hour. 

### 2.3. Cell Culture

Saos-2, a human osteosarcoma cell line, was obtained from Riken cell bank (Tsukuba, Japan). These human osteoblast-like cells (Saos-2) have been widely used as a model system for human osteoblastic cells in biomaterial studies [10, 11]. The cells were seeded on uncoated (*n* = 8) and MWCNT-COOH-coated zirconia discs (*n* = 8) and in culture dishes (*n* = 8) which served as controls, where *n* indicates the number of samples. The seeding density was 10^4^ cells/cm^2^, and they were grown at 37°C in 5% CO_2_ and 95% air environment in Dulbecco's Modified Eagle's Medium (Sigma Aldrich, St. Louise, Mo, USA) supplemented with 10% FBS (MP Biomedical, LLC, Tokyo, Japan) and 1% penicillin-streptomycin antibiotic mixture (MP Biomedical, LLC, Tokyo, Japan). The cell viability, proliferation, and morphology were evaluated after the cells were cultured for 0 (6 hours after seeding), 2, 4 and 6 days.

### 2.4. Proliferation Test

The proliferation of the cells was evaluated using a Cell Counting Kit-8 (CCK-8) (Sigma-Aldrich Chemie GmbH, Buchs, Switzerland), which is a highly water-soluble tetrazolium salt WST-8 assay; 50 *μ*L CCK-8 was added in the cell medium per well and incubated for 1 h at 37°C. Afterwards, 100 *μ*L of the supernatant of each well was removed into a 96-well plate, and the absorbance was measured at 450 nm using a microplate reader (Bio-Rad, Hercules, CA, USA).

### 2.5. Live or Dead Viability Test

The LIVE/DEAD viability/cytotoxicity kit for mammalian cells (Molecular Probes, Eugene, OR, USA) was used to test the cell viability. 150 *μ*L of the LIVE/DEAD assay reagent solution was added to the cultured surface of the disc samples. After 30 min incubation in room temperature, 10 *μ*L of the LIVE/DEAD assay reagent solution was added to a clean dish. The zirconia disc was mounted on the dish, and the cells were observed in a fluorescence microscope (Olympus, IX 81, Tokyo, Japan) immediately, and photographs were taken during the examination. 

### 2.6. Detachment Test

As trypsin-EDTA solution is generally used to detach cells, the cell adhesion was estimated by treatment with this solution. On day 6, the Saos-2 cells cultured on the MWCNT-coated and uncoated zirconia discs were treated with 0.02% trypsin-EDTA solution for 10 min. After detachment, the discs were observed using scanning electron microscopy (SEM: S-4800, Hitachi, Tokyo, Japan).

### 2.7. SEM Evaluation

The samples were rinsed with phosphate buffered saline (PBS) to remove nonadherent cells and chemically fixated in a solution of 2.5% glutaraldehyde for 24 h at 4°C. The disc samples were then dehydrated in a series of solutions with increasing ethanol concentrations (50%, 70%, 80%, 90%, 95%, and 100%) followed by critical-point drying at 37°C. Finally, the samples were coated with Pt-Pd alloys in an ion sputter (E-1030, Hitachi, Tokyo, Japan) and the morphology of the cells was examined by SEM (S-4800, Hitachi, Tokyo, Japan). 

## 3. Results

**Figure 2:**
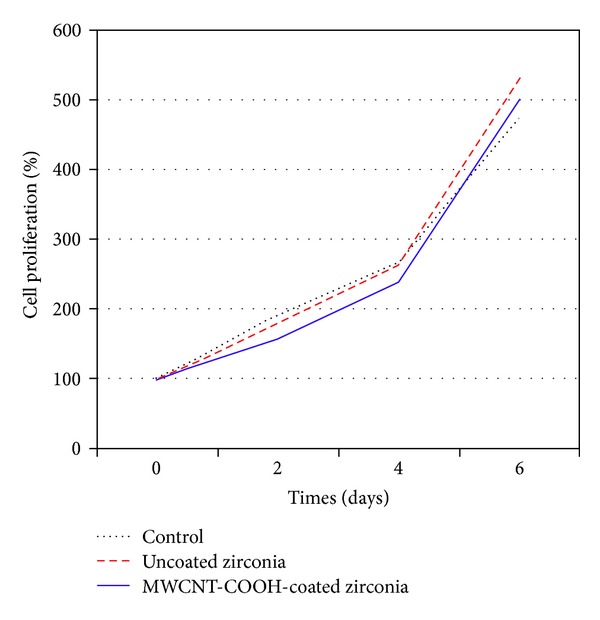
Proliferation curve for Saos-2 cells in culture dish (control), on uncoated zirconia and MWCNT-COOH-coated zirconia disc samples.

**Figure 3:**
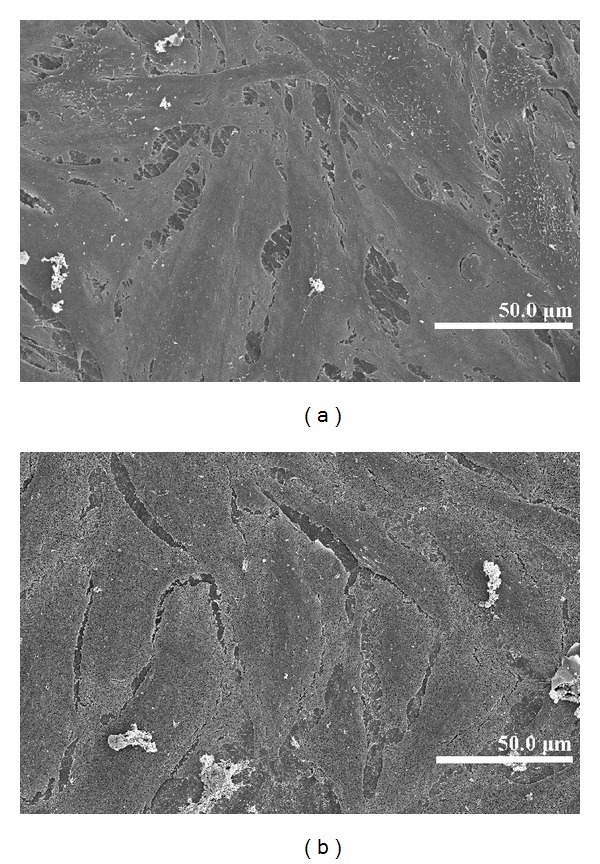
Cell morphology on uncoated (a) and MWCNT-COOH-coated (b) zirconia disc samples after 6 days observed by SEM, both were well covered with Saos-2 cells.

**Figure 4:**
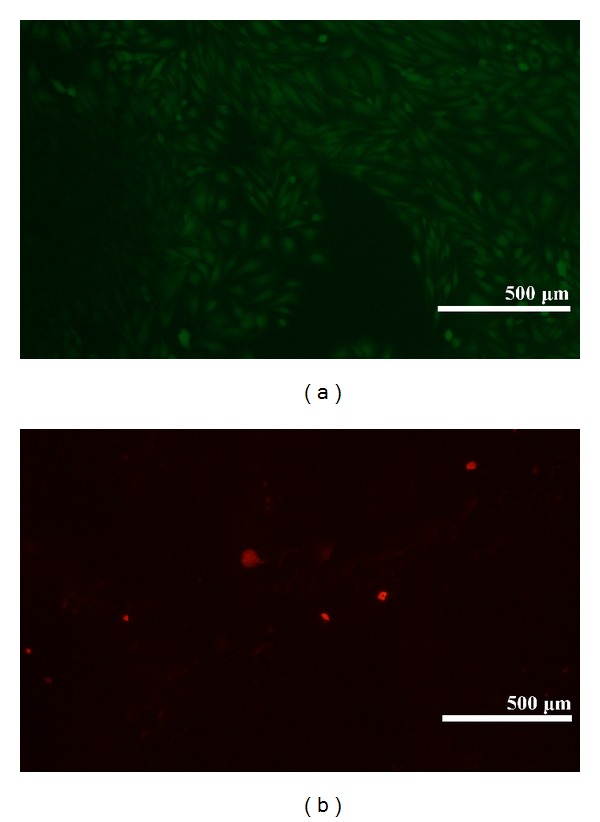
Visualization of cell viability on uncoated zirconia after 6 days by fluorescence microscope. High viability of the Saos-2 cells on uncoated zirconia is indicated. The green colored cells (calcein AM) indicate viable cells, and the red colored cells (ethidium homodimer-1) indicate dead cells.

**Figure 5:**
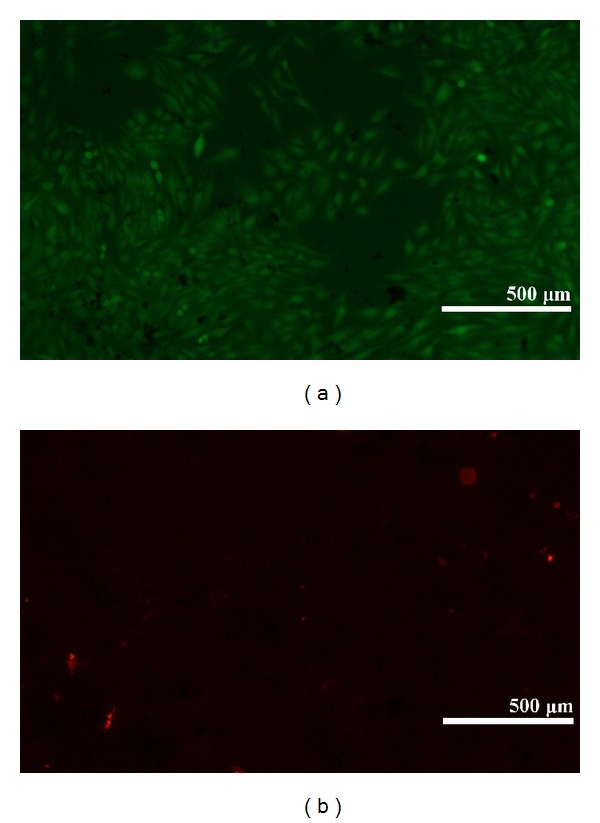
Visualization of cell viability on MWCNT-COOH-coated zirconia after 6 days by fluorescence microscope. High viability of the Saos-2 cells on MWCNT-COOH-coated zirconia is indicated. The green colored cells (calcein AM) indicate viable cells, and the red colored cells (ethidium homodimer-1) indicate dead cells.

**Figure 6:**
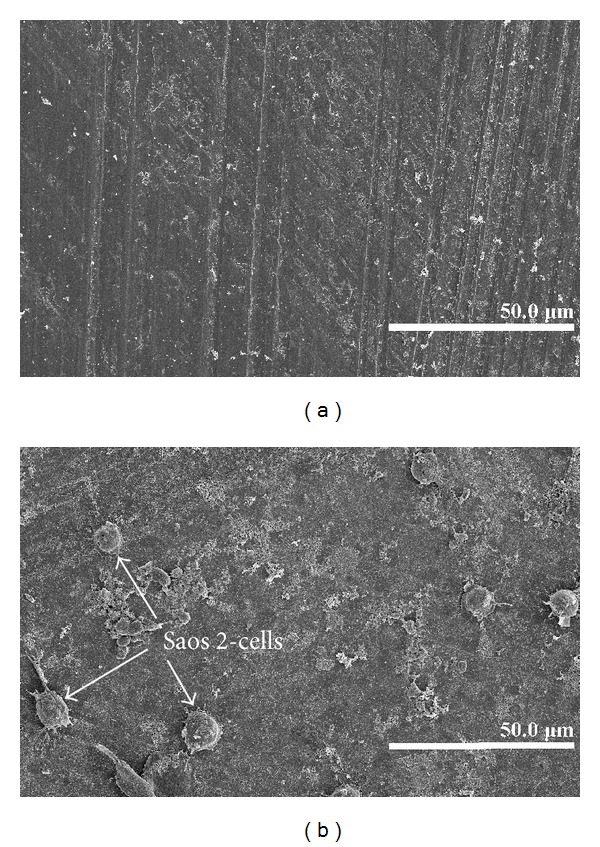
SEM images of Saos-2 cells on uncoated (a) and MWCNT-COOH-coated zirconia (b) after detachment treatment with 0.02% trypsin-EDTA.

**Figure 7:**
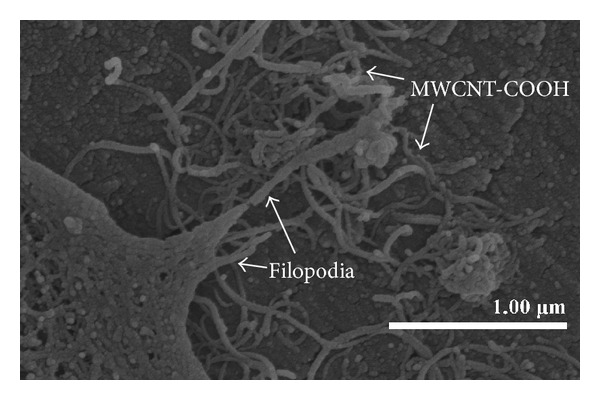
Enlargement of the periphery of a Saos-2 cell attached to the MWCNT-COOH-coated zirconia by SEM. The filopodia are well attached and elongated towards CNTs.

**Figure 8:**
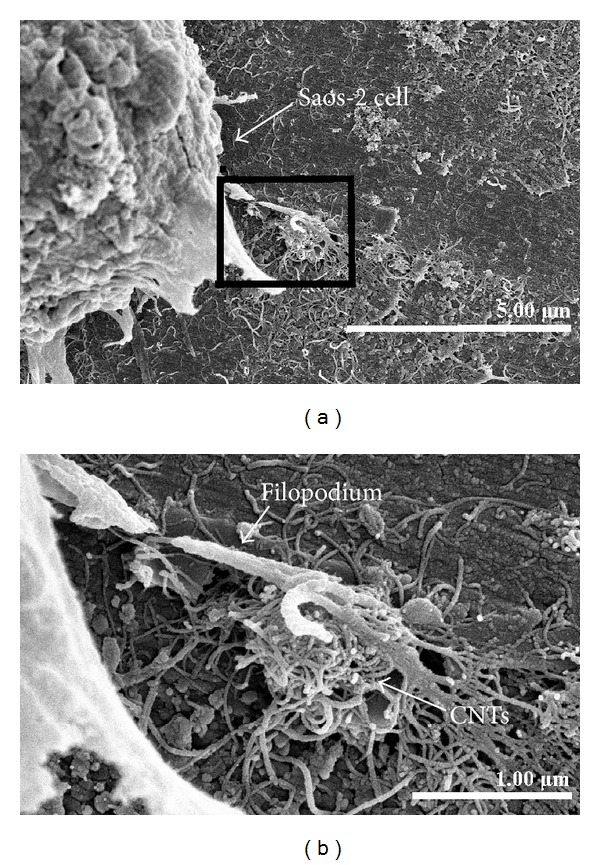
SEM of Saos-2 cell on MWCNT-COOH-coated zirconia discs after treated with trypsin-EDTA (a) and its enlargement (b). One filopodium is still attached with a cluster of agglomeration of CNTs (a, b). Breakage of the filopodium could be due to the dehydration during the sample preparative process for SEM observation.

A good covering of MWCNT-COOH could be observed on the coated zirconia discs (Figure 1). The cells were well attached both to the uncoated and MWCNT-COOH-coated zirconia samples. The proliferation curve for the control, uncoated and MWCNT-COOH-coated zirconia is similar in appearance (Figure 2). SEM analysis of the cell morphology showed that the Saos-2 cells were flattened and well attached to the surface of both the uncoated and MWCNT-COOH-coated zirconia with numerous filopodia and microvillosities, which are parts of the normal morphology of this kind of cell (Figure 3). High viability of Saos-2 cells was seen both on uncoated and MWCNT-COOH-coated zirconia (Figures 4 and 5). After application of trypsin-EDTA to measure cell detachment, a number of Saos-2 cells could still be observed on MWCNT-COOH-coated zirconia, whereas no cells were seen on the uncoated zirconia (Figure 6). In Figure 7 example of a Saos-2 cell attached to the MWCNT-COOH-coated zirconia is shown, and the filopodia seem to be well attached and elongated towards CNTs. In Figure 8 a and b example of a Saos-2 cell on a MWCNT-COOH-coated zirconia disc after treated with trypsin-EDTA is presented. One filopodium is still attached with a cluster of agglomeration of CNTs (Figure 8). 

## 4. Discussion

### 4.1. Osseointegration of Zirconia Implants

Fracture and/or loss of dental implants cause patient discomfort and often clinical bone loss. It is therefore important that dental implants possess good mechanical properties and osseointegration capability. As mentioned earlier, sandblasting is often performed on zirconia implant surfaces to increase the surface area in order to improve their osseointegration ability [3]. However, sandblasting can also introduce defects on the implant surface, which act as stress concentration sites and subsequently cause fracture of the implants [1]. In addition, sandblasting could have a negative impact on other material properties in zirconia ceramics such as phase transformation and Weibull modulus [12]. 

### 4.2. CNTs

Application of CNTs could conceivably be an alternative to sandblasting achieving sufficient surface roughness on the implants. In a previous study by Terada et al. [13], 100 ppm MWCNT-COOH was used to coat titanium, which led to an increase in surface roughness from Ra = 0.05 ± 0.01 *μ*m to Ra = 0.13 ± 0.01 *μ*m. A concentration of 100 ppm was, therefore, also used in the present study to coat the zirconia ceramic discs. 

Application of CNTs as a biomaterial has previously been debated [6], mostly because CNTs are very small in size and thus capable of entering the human body by inhalation, ingestion, and/or skin penetration and of interacting with intracellular structures [14]. Moreover, impurity in CNTs has been pointed out as one of the most important factors leading to toxicity, especially catalyst metal contaminants such as Fe, Y, Ni, Mo, and Co, amorphous carbon, and other carbon nanomaterials [15]. For this reason, the MWCNTs in the present study were purified by heating to remove amorphous carbon and by acid treatment to remove the metal contaminants and minimize cytotoxicity. In the present study, MWCNT-COOH-coated zirconia, cell culture dish, and uncoated zirconia all indicated a good proliferation of Saos-2 cells (Figure 2). Our observation of this good proliferation of Saos-2 cells on CNT coating is in agreement with the good proliferation of MC3T3-E1 cells on CNT-coated titanium in a previous study by Terada et al. [13]. Furthermore, MWCNT-COOH coating indicated no negative effect on the cell viability (Figure 5) and morphology (Figure 3). Thus, these results show that MWCNT-COOH coating of the zirconia discs did not cause any acute toxicity in vitro. 

### 4.3. Functionalization of CNTs

Functionalization of the MWCNTs is of importance for providing dispersion of CNTs in an aqueous solution [7]. The MWCNTs used in the present study were produced using the chemical vapor deposition method, implying that these synthesized CNTs should have open ends and be highly susceptible to chemical reaction [16]. Peroxide was utilized for functionalization of the MWCNTs to create carboxyl groups on the surface of MWCNTs, which could subsequently be dispersed in aqueous solutions [9]. These methods have been applied in a number of earlier studies [13, 17, 18], and good dispersion results have been reported with the methods [17]. In the present study, the MWCNTs were dispersed in alcohol into a homogenous black solution with sonification, so that after the alcohol had evaporated the zirconia surface was left coated with MWCNT-COOH. The samples were then rinsed with distilled water. The MWCNT-COOH seemed to be well attached to the surface of the zirconia discs throughout the cell culture procedure (Figure 7). 

### 4.4. Binding of Osteoblast-Like Cells with CNTs

Using of trypsin-EDTA is the most common method to detach cells from the cultured surface. It was shown in the present work that more cells were attached to the MWCNT-COOH-coated zirconia surface compared to the uncoated samples, which indicates preferable cell adhesion on the MWCNT-COOH-coated zirconia surface. In Figure 8, a round shaped Saos-2 cell can be observed. The rounded shape of the cell may have occurred because it reacted well with trypsin-EDTA and levitated from the disc surface. One of the filopodia, however, was still attached with an agglomerated cluster of MWCNT-COOH, which was one of the reasons why the Saos-2 cell was still attached to the surface of the MWCNT-COOH-coated zirconia disc. Matsuoka et al. [19] reported that the MWCNTs promote cell adhesiveness of Saos-2 cells due to the rougher topography of the surface of the MWCNTs coating. Firkowska et al. [20] reported that strong adhesion of osteoblasts to MWCNT-based substrates is correlated to a highly expressed focal adhesion of cells responding to nanoscale topography. These reports support the findings in the present study that MWCNT-COOH coating could improve cell adhesion on the substrate. In conclusion, MWCNT-COOH coating on zirconia seems to have no negative effects on cell viability, proliferation, or morphology in vitro. In addition, better attachment of osteoblast-like cells was found on the MWCNT-COOH-coated zirconia discs than on the uncoated ones. Thus, MWCNT-COOH seems to be a promising coating material for use with zirconia-based ceramic surfaces to increase the roughness and thereby enhance the osseointegration of zirconia implants.
